# Heat Stress Affects Pi-related Genes Expression and Inorganic Phosphate Deposition/Accumulation in Barley

**DOI:** 10.3389/fpls.2016.00926

**Published:** 2016-06-24

**Authors:** Andrzej Pacak, Maria Barciszewska-Pacak, Aleksandra Swida-Barteczka, Katarzyna Kruszka, Pawel Sega, Kaja Milanowska, Iver Jakobsen, Artur Jarmolowski, Zofia Szweykowska-Kulinska

**Affiliations:** ^1^Department of Gene Expression, Institute of Molecular Biology and Biotechnology, Faculty of Biology, Adam Mickiewicz University in PoznanPoznan, Poland; ^2^Department of Plant and Environmental Sciences, Faculty of Science, University of CopenhagenCopenhagen, Denmark

**Keywords:** barley, Pi transporters, PHO2, phosphate, high temperature, abiotic stress

## Abstract

Phosphorus (P) in plants is taken from soil as an inorganic phosphate (Pi) and is one of the most important macroelements in growth and development. Plants actively react to Pi starvation by the induced expression of Pi transporters, *MIR399, MIR827*, and miR399 molecular sponge – *IPS1* genes and by the decreased expression of the ubiquitin-conjugating enzyme E2 (*PHOSPHATE2* – *PHO2*) and Pi sensing and transport *SPX-MFS* genes. The PHO2 protein is involved in the degradation of Pi transporters PHT1;1 (from soil to roots) and PHO1 (from roots to shoots). The decreased expression of *PHO2* leads to Pi accumulation in shoots. In contrast, the *pho1* mutant shows a decreased level of Pi concentration in shoots. Finally, Pi starvation leads to decreased Pi concentration in all plant tissues. Little is known about plant Pi homeostasis in other abiotic stress conditions. We found that, during the first hour of heat stress, Pi accumulated in barley shoots but not in the roots, and transcriptomic data analysis as well as RT-qPCR led us to propose an explanation for this phenomenon. Pi transport inhibition from soil to roots is balanced by lower Pi eﬄux from roots to shoots directed by the PHO1 transporter. In shoots, the *PHO2* mRNA level is decreased, leading to an increased Pi level. We concluded that Pi homeostasis in barley during heat stress is maintained by dynamic changes in Pi-related genes expression.

## Introduction

Phosphorus (P) is an essential macronutrient for plant growth and development. It is a component of vital molecules such as ATP, DNA, RNA, phospholipids, and phosphorylated sugars, making it crucial to cellular metabolism (Huang et al., 2008), energy conversion, and cell structure. Plants take up P solely as inorganic phosphate ions (Pi) from low concentrations in the soil solution. Most soil P is adsorbed, immobilized in organic matter, or precipitated as minerals (Poirier and Bucher, 2002).

Plants have a range of strategies to cope with a limiting Pi supply, including (i) favoring root growth over shoot growth, (ii) increasing the activity of high-affinity Pi transporters, (iii) exuding protons and organic anions to liberate Pi in the rhizosphere, and (iv) secreting acid phosphatases and ribonucleases to release Pi from organic compounds (Nilsson et al., 2007). Phosphate-deficient plants mobilize Pi from different subcellular compartments and organs and increase their Pi use efficiency by promoting metabolic bypasses that favor Pi-releasing reactions (Nilsson et al., 2007; Huang et al., 2008).

The uptake and redistribution of Pi is mediated by many different Pi transport proteins. These transporters have been classified into four families (dependent on their cellular location): PHT1 (plasma membrane), PHT2 (plastid inner envelope), PHT3 (mitochondrial inner membrane), and PHT4 (plastid inner envelope) (Karandashov and Bucher, 2005; Guo et al., 2008). The main role in Pi transportation from soil to plant roots is played by proteins belonging to the PHT1 Pi transporter family. Ten *PHT1* genes have been identified in barley, and some of them have almost identical sequences; i.e., *HvPHT1;1*, and its paralogs *PHT1;2, PHT1;9*, and *PHT1;10*. These paralogs are expressed only in roots and are highly responsive to Pi limitation (Huang et al., 2011). *HvPht1;6* is expressed in roots and in the older leaves of shoots (Preuss et al., 2010). The *HvPHT1;8* gene is activated in the presence of arbuscular mycorrhizal fungi (Christophersen et al., 2009). In *Arabidopsis* PHT1;1 and PHT1;4, members of the PHT1 Pi transporter family play a central role in Pi acquisition (Shin et al., 2004). Another Pi transporter gene (*PHO1*) is primarily expressed in the root stelar cells and is involved in Pi loading into the xylem (Liu et al., 2014; Wege et al., 2016), and the *pho1* mutant shows a low Pi level in shoots (Liu et al., 2014). Rice PHO1;2 is involved in the long-distance transport of Pi from roots to shoots (Wang et al., 2015). SPX-MFS proteins are, in turn, involved in Pi sensing and transport. In rice, OsSPX-MFS1 and OsSPX-MFS2 are targeted by Pi-responsive miR827 (Wang et al., 2012). This microRNA in *Arabidopsis* targets *NLA* (Nitrogen Limitation Adaptation) mRNAs that encode E3 ubiquitin ligase. Recently, it was shown that NLA protein works together with PHO2 (PHOSPHATE2, ubiquitin-conjugating enzyme E2), and they are both responsible for PHT1;4 degradation via the 26S proteasome (Park et al., 2014). PHO2 is involved in defining cellular Pi homeostasis and is responsible for the ubiquitin transfer to the protein substrate via the E3 enzyme (ubiquitin-protein ligase) (Smalle and Vierstra, 2004). The PHO2 protein itself is involved in the degradation of the PHT1;1, PHT1;2, PHT1;3, PHT1;4, PHO1, and PHF1 (PHOSPHATE TRANSPORTER TRAFFIC FACILITATOR1) (Liu et al., 2012; Huang et al., 2013). Thus, it is a general negative regulator of Pi uptake. The response of plants to Pi deprivation requires specific sensing mechanisms to monitor Pi status as well as signaling mechanisms leading to transcriptional and post-transcriptional regulation. PHR1 from *Arabidopsis thaliana* is a MYB-related transcription factor (TF) that regulates the expression of Pi-starvation induced genes through binding to the *P1BS* (PHR1-binding sequence) sequence, a DNA motif in the promoters of many Pi-related genes (Bari et al., 2006; Sobkowiak et al., 2012). There are three P1BS-like elements within the barley *PHT1;1* promoter that are potentially recognized by PHR1 (Schunmann et al., 2004a). Moreover, in the *Arabidopsis IPS1* promoter, there are two *P1BS* motifs (Bustos et al., 2010). In rice, PHR1 and PHR2 (an ortholog of AtPHR1) are involved in Pi-related gene regulation (Wang et al., 2014). Although AtPHR1 and OsPHR2 regulate the expression of Pi-related genes, they are rather stably expressed and do not belong to the group of Pi-stress responsive genes (Wang et al., 2014). The Pi homeostasis regulatory system also comprises the actions of microRNA399 and microRNA827 (Bari et al., 2006; Hackenberg et al., 2013). miR399 guides the RISC complex to target *PHO2* mRNA. Furthermore, a riboregulator *IPS1* can bind and quench miR399 activity because it is not cleaved by the miRNA due to the base pair mismatches, which prevents *PHO2* mRNA against complete degradation (Franco-Zorrilla et al., 2007). Also, genes such as *Arabidopsis SIZ1* (SIZ/PIAS-type SUMO (Small Ubiquitin-related Modifier) E3 ligase) are involved in Pi response (Miura et al., 2005). Rice mutant *Ossiz1* shows an increase of Pi concentration in both root and shoot tissue (Wang et al., 2015). In *Arabidopsis*, SIZ1 is responsible for the SUMOylation of PHR1 (Miura et al., 2005). Although *OsSIZ1* itself is not very responsive to Pi starvation, it regulates the expression of other Pi-related genes, like *MIR399a, PHO2, PHR2*, or Pi transporters (Wang et al., 2015).

Heat stress has a big impact on plant growth and development. High temperature affects flowering time, reduces number, and leads to a reduction of size as well as an increase of deformity in floral organs (Zinn et al., 2010). Heat stress is also responsible for changing gene expression, including microRNAs and (as a consequence) their target mRNA levels. Barley microRNAs like miR160a, 166a, 167h, and 5175a are upregulated in heat stress, and consequently, the appropriate target genes are downregulated (Kruszka et al., 2014).

Transcriptomic data analysis derived from 1-h heat-stressed plants shows dramatic changes in Pi-related gene expression in roots as compared to unstressed controls. Based on their expression changes, a network of particular gene expression regulation and its influence on Pi homeostasis can be proposed. The expression of the *HvPHT1;1, HvPHT1;4*, and *HvPHT1;6* genes is downregulated in roots, which therefore should have decreased Pi concentrations. However, this is not the case; possibly, because the expression of the *PHO1* gene is concurrently decreased in the root. A lower PHO1 level can lead to lower Pi eﬄux from roots to shoots. Pi concentration in roots (in control and in heat treated plants) was always on the same level with one exception at 4 h time-point. The observed increased Pi concentration was correlated with significant *PHT1;4* up-regulation. Moreover, transcriptomic data reveals that *PHO2* and *SPX-MFS* are downregulated in roots, but not *PHR1, PHR2*, nor *SIZ1*. In heat treated shoots at two time-points: 1 h and 12 h we observed increased Pi concentration. It was correlated with *PHO2* and *PHT1;6* genes expression profile changes. At these time-points, the barley shoots showed increased Pi concentration; this agrees with previous data showing that *PHO2* silencing leads to an increase of Pi concentration in barley shoots (Pacak et al., 2010). Since we detected neither mature miR399 nor significant changes in the expression of mature miR827 in barley shoots, we concluded that the changes observed resulted from transcriptional regulation. The transcriptomic data was mostly confirmed by the RT-qPCR results. The changes in Pi concentration in barley shoots at 1 and 12 h heat time-points are abolished in other hours of heat stress, leading to Pi-concentration stability. Summarizing, the changes in expression of Pi-related genes after heat stress are not chaotic but strictly controlled, regarding what is necessary for the plant to maintain Pi homeostasis. During Pi re-supply, in barley roots we observed significant downregulation of the *PHT1;1, PHT1;4, PHT1;6, PHO1, PHO2* but not *PHR1, SPX-MFS*. In contrast to heat stress condition, at 1 h after Pi re-supply we observed increased expression of *PHO1* in Pi-resupplied plants compared to the control ones. We concluded that fine-tuning changes of *PHO1* expression in barley roots prevents Pi over-accumulation.

## Materials and Methods

### Plant Growth Conditions

In the heat experiment, barley plants cultivar Rolap were grown under conditions as previously described (Kruszka et al., 2013), and the heat stress conditions were established also as previously described (Kruszka et al., 2014) – except that the soil water content was kept at 70% to prevent drought stress. The soil/send mixture (7:2) was amended with 35 ml of a medium containing: 28 mM NH_4_NO_3_, 20 mM KH_2_PO_4_, 4 mM K_2_SO_4_, 16 mM MgSO_4_x7H_2_O, 53 μM H_3_BO_3_, 8 μM CuSO_4_, 4 μM MnSO_4_xH_2_O, and 120 μM FeCl_3_x6H_2_O. Two-week-old barley plants exposed 48 hours to heat stress were harvested at 1, 3, 4, 5, 6, 7, 8, 12, 24, and 48 h after the initiation of 35.5°C heat stress. Unstressed control plants were also grown (at 22°C). In the P stress experiment, barley plants cultivars Rolap and Black Hulless were grown at different Pi regimes in a 7:2 (cv. Rolap) and 1:1 (cv. Black Hulless) (w/w) soil-sand mixture containing 8 mg P kg^-1^ soil (extraction by 0.5 M NaHCO_3_, (Olsen et al., 1954)). The soil received either nil or 60 mg P kg^-1^ that was mixed into the soil as KH_2_PO_4_. Plants were grown under controlled conditions at 16 h/8 h day/night and 22°C (cv. Rolap) or 21°C/15°C (cv. Black Hulless). For barley cv. Rolap at 2 weeks after sowing, half of the plants at nil P received 60 mg of P/kg soil as a KH_2_PO_4_ solution. Three pots of each P treatment were harvested sequentially as follows: P sufficiency (control), P limitation, P replenishment, +1 h, +3 h, +12 h, +24 h and +48 h. Each pot contained two barley plants. For barley cv. Black Hulless at both 10 and 17 days after sowing, each pot received 45 mg N/kg as an NH_4_NO_3_ solution. At 20 days after sowing, half of the plants at nil P received 60 mg of P/kg soil as a KH_2_PO_4_ solution. Three pots of each P treatment were harvested sequentially as follows: P sufficiency and P limitation, 10 days post sowing (das), 15 das, 20 das +1 h, 21, and 23 das; + P replenishment, 20 das +1 h, 21 and 23 das. Each pot contained three barley plants.

### Phosphate Measurement

The level of Pi in plant root and shoot samples was monitored by an inorganic P assay utilizing malachite-green/ammonium molybdate in 4 M HCl and 34% natrium citrate*2H_2_O solutions (Lanzetta et al., 1979). Fifty milli gram of liquid *N*_2_ frozen plant tissue was homogenized in a glass homogenizer in 1 ml of ice-cold 1 M HCl. After centrifugation, the supernatants were used for a micro-plate assay at room temperature. The standard curve was constructed from a P-stock solution of 1 nmol/μl KH_2_PO_4_ in two replicates. The samples were measured in two technical and three biological replicates. The OD_660_ was read (Müller et al., 2004) using either a SpectraMax M5 Microplate Reader (Molecular Devices, LLC, USA) or Infinite F200 Pro (TECAN, Switzerland).

### RNA Isolation

For the barley heat stress experiment, RNA isolation procedure was described previously (Kruszka et al., 2013, 2014). In brief, the RNA isolation method utilized a mixture containing a 38% v/v phenol solution saturated with 0.1 M sodium acetate (Roti Aqua Phenol, Roth, Karlsruhe, Germany), supplemented with 0.8 M guanidine thiocyanate, 0.4 M ammonium thiocyanate, 0.1 M sodium acetate, 5% v/v glycerol, 0.5% sodium lauroylsarcosine, and 5 mM EDTA. To remove polysaccharides, the Ambion Plant RNA Isolation Aid (Thermo Fisher Scientific, Lithuania) was added, with the resulting supernatant being purified using a Direct-zol^TM^ RNA MiniPrep kit according to the manufacturer’s protocol (ZYMO RESEARCH, The Epigenetics Company, USA).

### cDNA Synthesis and PCR Reactions

cDNAs used in RT-PCR and RT-qPCR analysis were synthesized using an oligo(dT)_18_ primer, SuperScript III Reverse Transcriptase (Invitrogen, USA) and 3 μg of Turbo DNase (Ambion, Lithuania) treated RNA as a template. cDNAs were diluted four times, and 1 μl was used in RT-qPCR and diluted five times, with 2 μl used for RT–PCR amplification. PCR amplification of either an ubiquitin or ADP cDNA fragment were used as a positive control and control of the sample loading. The purity of cDNA samples containing no genomic DNA was controlled by PCR amplification of a barley *HvPHT1;1*; GenBank accession no. AF543197.1 promoter fragment (Schunmann et al., 2004b; Kruszka et al., 2014).

### PCR Amplification

Primers for RT-qPCR amplification of *HvPHT1;1/HvPHT1;9, PHR1, PHO2, IPS1*, and ubiquitin were previously described (Pacak et al., 2010). The rest of the used primers are summarized in **Table 1**.

**Table 1:** Sequences of used primers.

Name	Sequence 5′ to 3′	Task
APO175	GGGATAACATCCCTGCTTCTGGTAGTC	3′ RACE, *PHR1*
APO176	CATCCCTGCTTCTGGTAGTCAGATCC	3′ RACE, *PHR1*
APO177	ATGAGATGAAGCTGGCTGGTTGAC	5′ RACE, *PHR1*
APO178	GAATTGGAAGGCTGAACCATGGAC	5′ RACE, *PHR1*
APO273	CCATGATGTACATTTTGCACAAACCACCTA	3′ RACE, *PHO2*
APO274	CACGTACATATCAGATGATGGCTGCAAGAG	3′ RACE, *PHO2*
APOYY1	CTCGGTCACTGAAGAAGAGGGAGCA	5′ RACE, *PHO2*
APOYY2	CCCCTCCAAGAAACGACCAGC	5′ RACE, *PHO2*
APO392	TGTAAAACCACGGCACAGAA	PCR, *ADP*
APO410	CTTGAAGCGTATCGAGGAC	PCR, *ADP*
APO623	AAGGTCAAGGCAAGGAGGAA	RT-qPCR, pri-miR399c
APO624	CTGCCAATAAAGAGGAGCCC	RT-qPCR, pri-miR399c
APO627	CAGGGCAACTCTCCTTTGGCA	Probe, miR399c
APO697	TGTTTGCTGATGGTCATCTAA	Probe for miR827
APO698	GCAACTCCCTCTTCGTGCT	RT-qPCR, *Pht1;4*
APO699	TTCGTGCCTGGTCCTCGTT	RT-qPCR, *Pht1;4*
APO700	CAAAGGCAACTGACGAGTGA	RT-qPCR, *PHR1*
APO701	ATGACTGAGGAGCGAAAGGA	RT-qPCR, *PHR1*
APO702	TTGAAGTCCGCAGATCACAG	RT-qPCR, *NLA*
APO703	ACTCGCTCCATCTGCATTCT	RT-qPCR, *NLA*
APO704	GTAGGCCTGACCTGCATCTG	RT-qPCR, *SPX-MFS*
APO705	ACCAATGGCTGAGGAAACAG	RT-qPCR, *SPX-MFS*
APO706	Described by (Huang et al., 2011)	RT-qPCR, *Pht1;6*
APO707	Described by (Huang et al., 2011)	RT-qPCR, *Pht1;6*
APO708	Described by (Huang et al., 2011)	RT-qPCR, *Pht1;1*
APO709	Described by (Huang et al., 2011)	RT-qPCR, *Pht1;1*
APO739	ATGGGTGCAGTTCTCTGAATG	RT-qPCR, *PHO1*
APO740	CTGAAGAACCTTGTCAGACG	RT-qPCR, *PHO1*
APO741	AACTTCATGAGCGTTTTGTGG	RT-qPCR, *PHR2*
APO742	TCGGGCAGTTCTGTACTTCT	RT-qPCR, *PHR2*
APO743	AGGATGCTCTTGCTCGTGTT	RT-qPCR, *SIZ1*
APO744	CAGCGGTCTTCATTCTGGAT	RT-qPCR, *SIZ1*

For RT-PCR, “Touchdown” PCR was applied (Tolia and Joshua-Tor, 2006). RT-qPCR was performed as previously described (Kruszka et al., 2013).

RACE and RLM-RACE were performed using a SMARTer^TM^ RACE cDNA Amplification Kit (Clontech, Mountain View, CA, USA) and GeneRacer kit (Invitrogen), respectively (according to the manufacturers’ protocol).

For RT-qPCR analysis, an Applied Biosystems 7900HT Fast Real-Time PCR System and SYBR Green Master Mix (Applied Biosystems) were used. Data was normalized to ADP levels. The results were presented as either log_10_2^(-ΔCt)^+7 or as fold changes as previously described (Kruszka et al., 2013). The *R*^2^ values of analyzed data (≥0.997) were calculated with LinRegPCR software (Ramakers et al., 2003). Three biological replications were analyzed.

### Northern Blot

Experiments were performed using either 50 μg RNA enriched in small RNA isolated from barley shoots (**Figure 6**), according to (Szarzynska et al., 2009; Kruszka et al., 2013) or 7.5 μg RNA enriched in small RNA isolated from barley roots (**Figure 10**), according to (Pall and Hamilton, 2008).

### Transcriptome Analysis

Total RNA was isolated using the procedure described above. Isolated RNA samples were Turbo DNAse (Ambion) treated and then phenol/chloroform purified. Three biological replications of barley shoots and two biological replications of barley roots harvested after 1 h of heat treatment were analyzed (as well as the control samples). Transcriptome libraries (strand-specific) were constructed and then sequenced by BGI Tech Solutions (Hong Kong) Co., Ltd. Clean reads were mapped to the reference barley genome ^1^ (Ensembl Plants, version: 082214v1) using TopHat2 software (Kim et al., 2013). Statistical analysis was performed to identify gene-expression differences between the samples studied (TEAM, 2010; Pinheiro et al., 2014). Numbers of reads for each gene mapped to the reference genome were counted using HT-Seq software (Anders et al., 2015). The script was run for each fastq file for every biological replicate from every condition. Afterward, all of the results were gathered in tables – one table per one comparison (e.g., a table with two biological replicates from roots treated with high temperature and two replicates from control roots). These tables were then fed in a DeSeq – R package suitable for analysis of differential gene expression based on the negative binomial distribution (Anders and Huber, 2010). The data used in this study have been deposited under NCBI GEO accession GSE82134.

### Gene Identification

Transcriptomic analysis met some difficulties caused by sequence similarities between gene paralogs. For instance, the MLOC_28370 gene encodes a 369AA protein identical to HvPHT1;1 (GenBank AAN37900.1; AF543197), HvPHT1;2 (AAO72434.1, AY187020), and HvPHT1;9 (GenBank CAP17759; AM904733), both on nucleotide and amino-acid levels. Thus, transcriptomic reads reflect expression of these three Pi transporters. To distinguish the expression of the *PHT1;1* gene from their paralogs particular Pi transporter, two sets of RT-qPCR primers were used for analysis. We analyzed both *PHT1;1/PHT1;9*, and concrete *PHT1;1* gene expression. We identified the putative barley *PHR1* gene by comparison with the well-known *Psr1* (Phosphate starvation response 1) and *PHR1* genes from *Chlamydomonas reinhardtii*, rice, and *Arabidopsis*. We found several barley ESTs (BU993345, BU970600 and BF621611). In further steps, these ESTs were clustered into one mRNA fragment. Based on this mRNA fragment sequence, a pair of primers was designed. The amplified PCR product (923 bp in length) – i.e., fragment of *HvPHR1* – was sequenced as previously described (Pacak et al., 2010). To determine full-length *HvPHR1*, 5′ and 3′ RACE experiments were conducted. The coding sequence had 1356 bp and can encode a 451AA sequence that has 99% identities to MLOC_5585. Barley *PHO1* – MLOC_12153.1 contains an amino acid sequence with 298/477 (62%) and 432/475 (91%) identity (blastp analysis) to the *Arabidopsis* PHO1 protein (GenBank NP_188985.2) and *Brachypodium distachyon* phosphate transporter PHO1-2 (GenBank XP_003570364.1), respectively. We found contig TCONS_00089457 that contains the mature barley miR399c sequence. The pre-miR399c sequence was identified in the transcriptomic data derived from heat-treated shoot tissue – TCONS_00089457. This sequence was mapped to morex_contig_188578 CAJW010188578 carma = 2HL (ipk-gatersleben database). The longest derived sequence that formed a stem-loop structure was used for construction of the pre-miR399c. Using 5′ RLM-RACE, two TSS of the barley *PHO2* gene were identified at positions 447916090 (+) and 447916332 (+) on the chromosome 1. Promoter region (1422 bp) upstream of the 2nd TSS (heterogenic) was used for promoter elements analysis. Later, a *PHO2* genomic sequence was establish based on 5′ and 3′ RACE analysis and morex_contig_38340 CAJW010038340 carma = 1H DNA sequences deposited at the ipk-gatersleben database.

### Software

The alignment and trees were constructed using CLC Main Workbench 7 software with the following parameters: (Kimura-2 parameters) and the consensus trees (neighbor-joining) with bootstrap value = 100 replications. Stem-loop structures were constructed using Folder Version 1.11 software with a RNAfold algorithm^2^. Significance of the results (*p*-value) was analyzed using student t-test. Statistical RT-qPCR analyses were performed using log_10_2^(-ΔCt)^+7 values.

### Database Searching

We searched the following databases: http://plants.ensembl.org/index.html, www.mirbase.org, and GenBank and IPK Gatersleben^3^. For promoter regulatory element analysis, a New PLACE (A Database of Plant *Cis*-acting Regulatory DNA Elements) database was used (Higo et al., 1999).

### Accession Numbers

Studied genes: Ensembl Plants database – Heat shock protein 90.1 – MLOC_5618, other accession numbers are deposited at **Table 2**. GenBank database: *HvPHT1;1* – AF543197, AAN37900.1; *HvPHT1;2* – AAO72434.1, AY187020; *HvPHT1;4* – AY187024.1, AAO72437.1; *HvPHT1;6* – FM866444.1, CAS02288; *HvPHT1;9* – AM904733, CAP17759; *HvPHR1* – GQ337895, ACT34981; *HvPHR2* – AK363485.1, BAJ94688.1; *HvPHO1* – AK364904.1, BAJ96107; *HvPHO2* – AK249253 (cv. Haruna Nijo); *HvPHO2* – GQ861514.1, ACV72276 (cv. Black Hulless); *OsPHO2* – LOC_Os05g48390 (AU032431); *AtPHO2* – AT2G33770.1; *HvNLA* – AK354779, BAJ85998.1; *HvIPS1* – GQ301528; *HvSIZ1* – AK366345.1, BAJ97548; *ADP* – ADP-ribosylation factor 1-like protein gene – AJ508228.2. The use of the Hsp17 gene as a “heat” stress gene marker (GenBank: AK252765) was described previously (Kruszka et al., 2014).

**Table 2:** Analyzed Pi-related genes with their Ensembl Plants database numbers and characteristics.

Gene	Ensembl Plants	Function	Notes
*PHT1;1*	MLOC_28370.1	Pi transporter	four paralogs in barley
*PHT1;4*	MLOC_6187	Pi transporter	
*PHT1;6*	MLOC_80912.2	Pi transporter	
*PHR1*	MLOC_5585	TF, ortholog of *OsPHR1*	
*PHR2*	MLOC_60198.1	TF, ortholog of *OsPHR2*	
*PHO1*	MLOC_12153.1	Pi transporter, homologous to rice *PHO1;2*	
*PHO2*	MLOC_53410.2	ubiquitin-conjugating enzyme E2, catalytic (UBCc) domain	targeted by miR399
*IPS1*	not identified in Ensembl Plants	binds miR399	position chr 4, 510585405-510586016
*NLA*	MLOC_52462.2	E3 ubiquitin-protein ligase, SPX_BAH1-like, RING domains	no miR827 binding site in barley
*SPX-MFS*	MLOC_57566.4	Pi sensing and transport SPX, MFS domains	targeted by miR827
*SIZ1*	MLOC_38182.4	PHR1 sumoylation	
Pre-miR399c	not identified in Ensembl Plants	miR399c targets *PHO2*	position chr 2, 558571927-558572025

*Barley genes *PHR1, PHR2, PHO1, NLA, SPX-MFS, SIZ1*, and *MIR399c* were identified based on their similarity to known orthologs and then allocated to a particular MLOC number.*

## Results

### Identification of Barley Pi-related Genes

**FIGURE 1:**
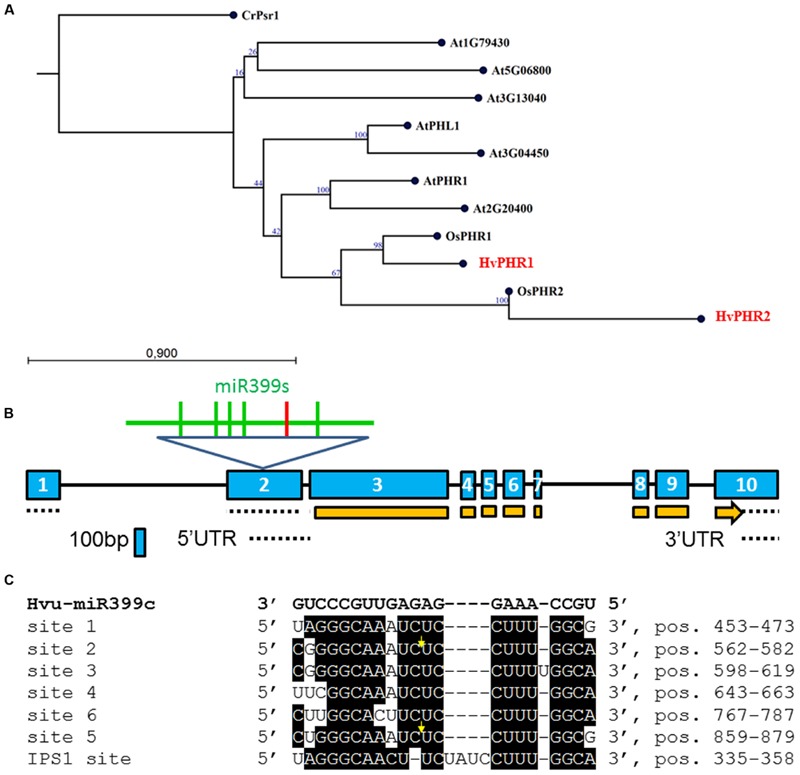
**(A)** Neighbor-Joining phylogram of the *PHR1*/*PHR2* transcription factor (TF) genes translated into amino acid sequences. Locus At5g06800 (protein – NP_196298.2); locus At5g29000 (PHR1-LIKE1 – PHL1, protein AAN86177.1); locus At2g20400 (protein – AAP04104.1); AtPHR1 – locus At4G28610 (protein – NP_194590); locus At1g79430 (protein – BAH19446); locus At3g13040 (protein – NP_974298); At3g04450 (protein – NP_187095.2); OsPHR1 – locus Os03g0329900 (protein – NP_001050006.1); OsPHR2 – locus Os07g0438800 (protein – BAG94425.1); CrPsr1 (nucleotide – XM_001700501, protein – XP_001700553); HvPHR1 (nucleotide – GQ337895, MLOC_5585, protein – ACT34981, MLOC_5585); HvPHR2 (MLOC_60198.1). Bootstrap value was calculated based on 100 replications; **(B)** Barley *PHO2* gene structure. Exons, introns, CDS and UTRs are depicted by blue boxes, black lines, orange boxes, and dotted lines, respectively. Within exon 2, six miR399 binding sites are depicted; the red vertical line represents binding site no 6; **(C)** Alignment of six miR399 binding sites in *HvPHO2* 5′UTR (according to AK249253) and one binding site in *HvIPS1* (GQ301528) recognized by miR399. Bold, black letters denote Hvu-miR399c sequence. Sites 1, 2, 3, 4, 5, and 6 are numbered starting from the 5′ end of the barley *PHO2* mRNA. Yellow arrows show cleavage position (Hackenberg et al., 2013). Site 6 (5′ CUUGGCACUUCUCCUUUGGCA 3′) has three mismatches to hvu-miR399d-3p (Hackenberg et al., 2013).

GenBank and Ensembl Plants databases were searched to identify barley Pi-related genes. Several Pi-related barley genes like Pi transporters (*PHT1;1, PHT1;4, PHT1;6*) and *PHR1, PHO2* were described previously (Schunmann et al., 2004a,b; Pacak et al., 2010; Preuss et al., 2010). **Table 2** shows all barley genes identified by us as well as other Pi-related barley genes. A phylogram was created to show the similarities of the identified barley PHR1/PHR2 TFs to the well-known PHR proteins from rice and *Arabidopsis* (**Figure 1A**) (Wang et al., 2014, 2015). Additionally, we have included the *Arabidopsis* PHR1-like protein (PHL1) and other MYB-CC proteins (Bustos et al., 2010).

**FIGURE 2:**
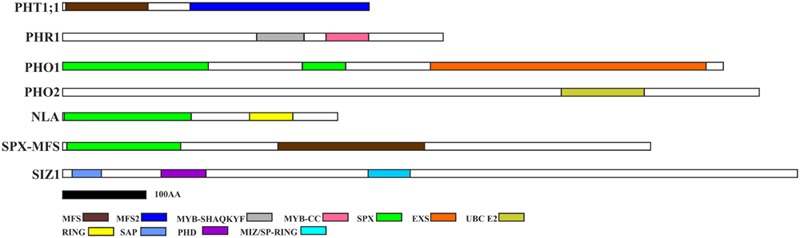
**Pi-related protein structures with depicted unique domains.** PHT1 proteins possess MFS (Major Facility Superfamily) domain characteristic for the secondary membrane transporter family (Wang et al., 2012); PHR1 contains LHEQLE motif, myb-like DNA-binding, and SHAQKYF class domains. PHO1 protein possesses SPX and EXS domains (Erd1-Xpr1-Syg1). PHO2 contains UBCc domain (ubiquitin conjugating enzyme); NLA protein possesses additional SPX domain of the E3 ubiquitin-protein ligase BAH1/NLA and RING-finger (Really Interested New Gene) domain; SPX-MFS has SPX and MFS domains; SIZ1 has following domains: SAP (SAF-A/B, Acinus and PIAS), plant homeodomain (PHD) finger, and MIZ/SP-RING (Msx – Interacting – Zinc finger) domain. Protein structures were constructed based on the appropriate protein sequences, whose accession numbers are deposited in **Table 2** – with the exception of PHO1 (protein – BAJ96107) and SIZ1 (protein – BAJ97548). The black bar represents 100 amino-acids.

Two splice variants of *PHO2* barley mRNA encoding 847AA and 544AA long proteins are deposited in Ensembl Plants. We compared *PHO2* CDS derived from MLOC_53410.2, with *PHO2* CDS derived from cv. Haruna Nijo, cv. Rolap, and cv. Black Hulless, and found that CDS from Haruna Nijo, and Rolap were identical, differing from Black Hulless in seven nucleotide substitutions (three synonymous and four non-synonymous mutations). The rice *PHO2* ortholog contains 11 exons and encodes an 876AA protein (Hu et al., 2011). In *Arabidopsis*, the *PHO2* gene has nine exons and encodes 907AA. The barley *PHO2* gene is located on chromosome 1, is 7320 bp in length, and contains ten exons (**Figure 1B**). The 5′ UTR of barley *PHO2* mRNA contains six potential miR399 recognition sites (**Figure 1C**). Only two of them were found to be cleaved in barley (Hackenberg et al., 2013). The schemes of the analyzed Pi-related proteins with depicted unique domains are presented in **Figure 2**.

**FIGURE 3:**
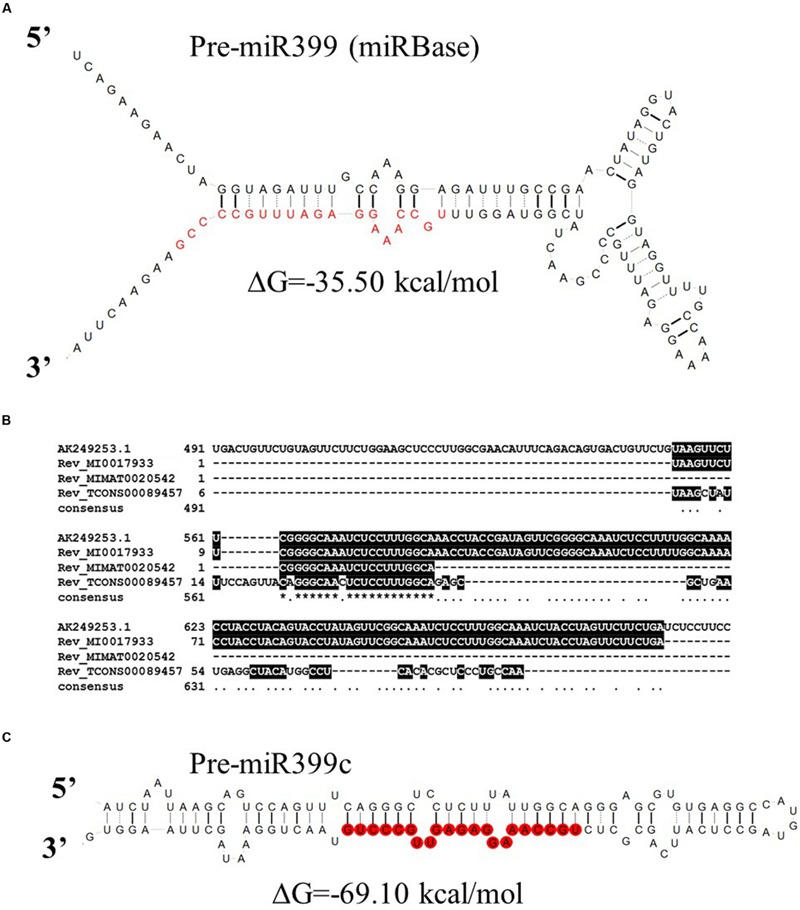
**Barley pre-miRmiR399 structure. (A)** Barley pre-miR399 stem-loop structure based on MI0017933 sequence – miRBase; **(B)** Alignment of the barley *PHO2* 5′ UTR and reverse complement miR399 related sequences. MI0017933 – miRBase, barley pre-miR399, MIMAT0020542 – miRBase barley microRNA399, fragment of TCONS_00089457 – Rolap, transcriptome identified pri-miR399c; **(C)** pre-miR399c stem-loop structure based on the TCONS_00089457 sequence (AGTCCAGTTTCAGGGCTCCTCTTTATTGGCAGGGAGCGTGTGAGGCCATGTAGCCTCATTCAGCGCTCTGCCAAAGGAGAGTTGCCCTGTAACTGGAAA).

Ten *MIR399* genes and one *MIR827* gene have been identified in the barley genome (Hackenberg et al., 2013). Both are induced by Pi deficiency (Hackenberg et al., 2013). In miRBase, there is only one pre-miR399 precursor available (MI0017933) from which mature miR399 could be diced out. This proposed pre-miR399 stem-loop structure does not contain the typical features of plant pre-miRNA (**Figure 3A**) (Bologna et al., 2009). Since it is possible that the miR399-3p sequence is unusually processed in barley shoots (Hackenberg et al., 2013), we decided to analyze it in more depth. We found that this sequence is, in fact, the reverse complement fragment of the *PHO2* 5′ UTR containing miR399 binding site no 2 (**Figure 3B**). We found a locus encoding the miR399c sequence and were able to fold it into a classical stem-loop structure with free energy ΔG = –69.10 kcal/mol (**Figure 3C**).

Our bioinformatics search allowed us to identify all known important players involved in Pi homeostasis maintenance in barley plants.

### Heat Stress Affects the Expression of Pi-related Genes

To test the heat-stress influence on Pi-related gene expression and Pi concentration, 2-week-old barley plants were stressed by exposure to a 35.5°C temperature for two days. Root and shoot samples were collected after 1, 3, 4, 5, 6, 7, 8, 12, 24, and 48 h.

**FIGURE 4:**
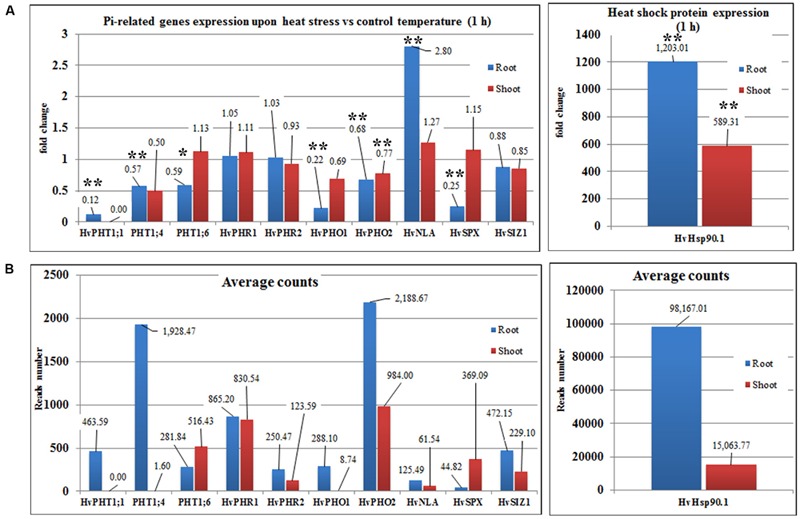
**Barley Pi-related gene expression based on NGS transcriptomic data under heat stress and control conditions (1-h duration). (A)** Fold changes of Pi-related gene expression in barley roots and shoots derived from heat stress compared to control conditions. **(B)** Average reads number of the Pi-related transcripts. Hsp90.1 was used as a control of heat-stress induction. ^∗^*p* < 0.05, ^∗∗^*p* < 0.005. Blue bars – gene expression in roots, red bars – gene expression in shoots, Transcriptomic analysis – *n* = 3 for shoots; *n* = 2 for roots.

**FIGURE 5:**
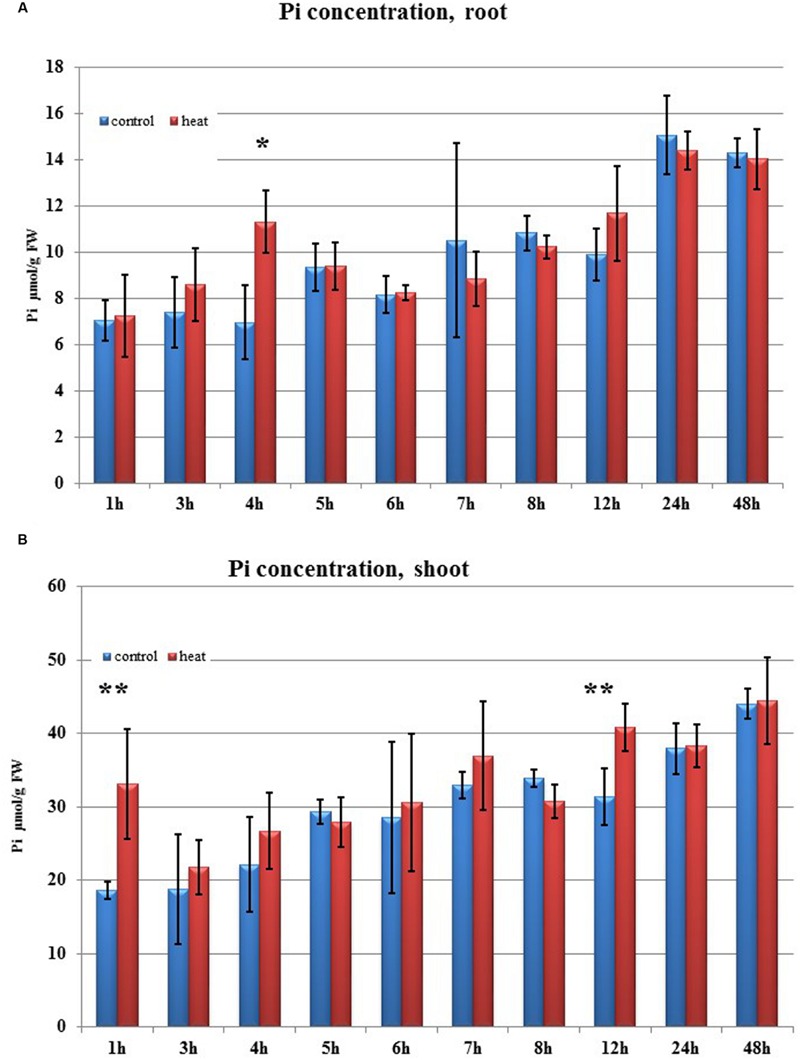
**Pi concentration. (A)** in barley roots and **(B)** in shoots after heat stress as compared to control plants. Blue and red bars represent control and heat-treated samples. ^∗^*p* < 0.05, ^∗∗^*p* < 0.005 (*t*-student test). Pi concentration *n* = 3, two technical replications.

**FIGURE 6:**
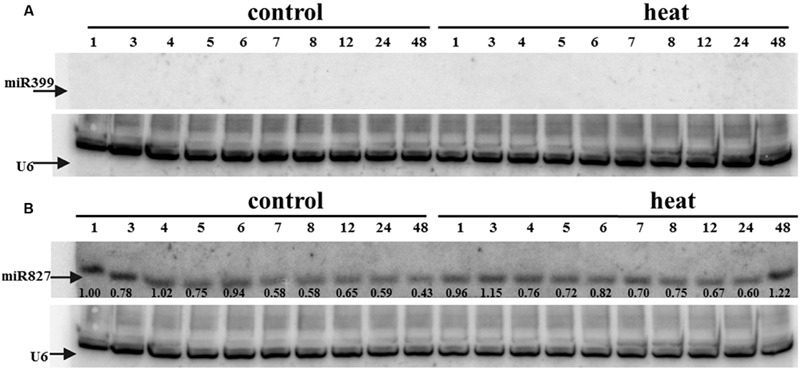
**(A)** miR399 and **(B)** miR827 expression levels in barley shoots. Northern blot was performed using probes against either miR399c or miR827. U6 was used as an RNA loading control.

**FIGURE 7:**
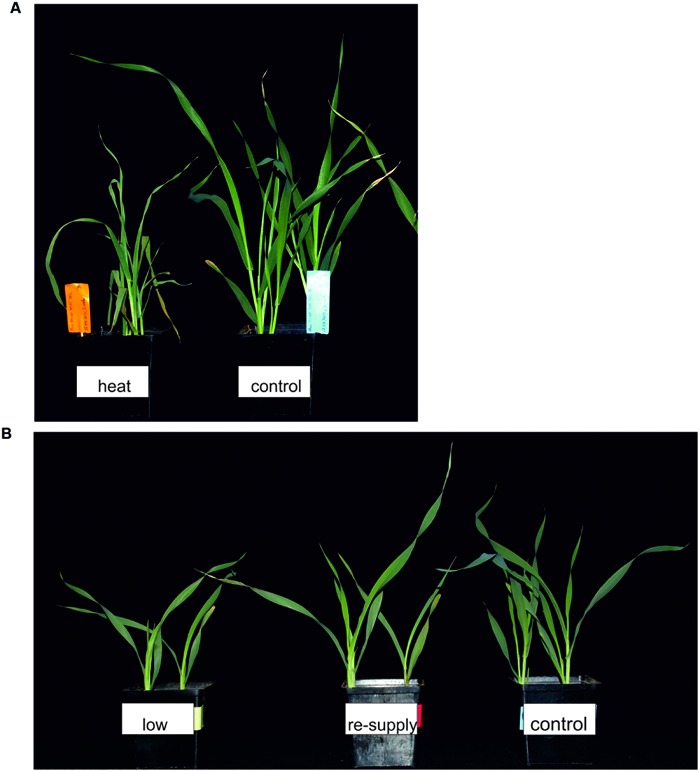
**Barley plants 48 h after treatment. (A)** 2-week old barley 48 h after heat treatment. **(B)** Phenotypes of 2-week old barley plants 48 h after Pi re-supply.

**FIGURE 8:**
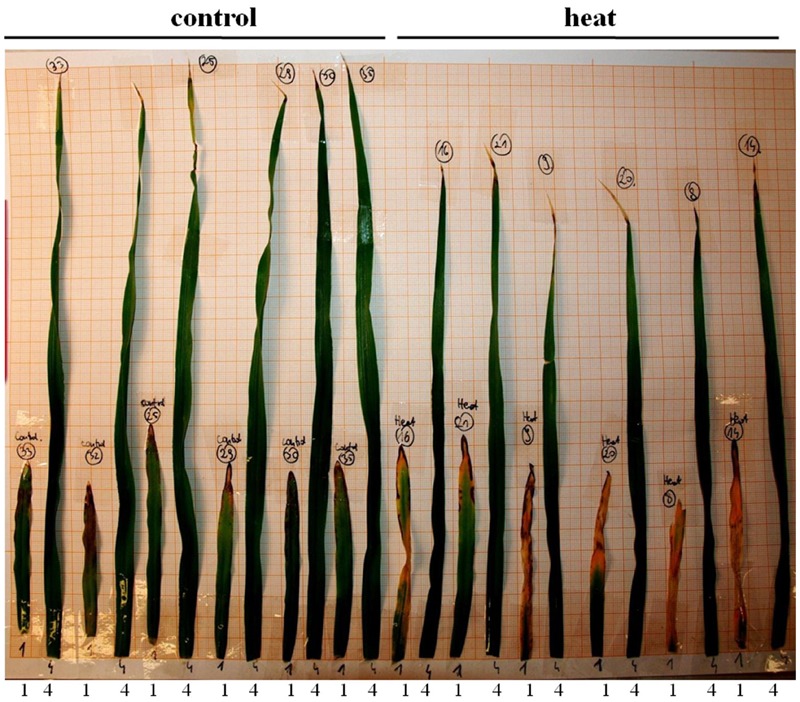
**Leaves of the barley plants one week after heat stress end.** The **(left)** represents control leaves; **(right)** heat-treated leaves. 1 and 4 denote leaf no. 1 and leaf no. 4, respectively.

Transcriptome deep sequencing was applied to RNA isolated from samples exposed to one hour of heat stress and from un-stressed control plants. Subsequently, our analysis was focused on the expression of Pi homeostasis maintenance-related genes. After one hour of heat stress, the expression of *PHT1;1, PHT1;4, PHT1;6, PHO1, PHO2*, and *SPX-MFS* genes was decreased in roots. Only the *NLA* gene showed an upregulated expression in the barley roots. The expression pattern of the *PHR1, PHR2*, and *SIZ1* genes was not significantly influenced. In contrast, the expression of most analyzed genes in shoots remained unchanged after one hour of heat stress. The only downregulated Pi-related gene was *PHO2* (**Figure 4**). The changes revealed in the transcriptomic data were confirmed by RT-qPCR (Supplementary Figures S1B–F,H–O,Q,R). The downregulated expression of genes in roots appeared to be permanent, as the *SPX-MFS* and *PHO2* genes remained downregulated after 48 h of heat stress. Since *PHT1;1* and *PHT1;9* CDSs have identical sequences, RT-qPCR designed for their 3′ UTR fragment amplification was performed, which allowed us to discriminate between the expression of these two genes. The data shows that both *PHT1;1/PHT1;9* (and also the *PHT1;6, PHO1, PHR2*, and *SIZ1* genes) stay at the lower expression level as compared to control plants. The *PHT1;4* gene shows a more-complex expression pattern; it was downregulated at 1 and 3 h, but up-regulation between 4 and 12 h of heat duration was also observed (Supplementary Figure S1C). In roots at 4 h time-point we observed the increased Pi concentration (**Figure 5A**). This correlated with *PHT1;4* significant up-regulation at this time-point. Since at 6 h time-point also *PHT1;4* gene expression was higher in heat stressed plants compared to the control ones, other factors could contribute to a mechanism of keeping the Pi concentration on the same level. The *NLA* gene was always upregulated, with the highest expression level at 24 h (Supplementary Figure S1H). Surprisingly, *IPS1* was also upregulated after 6 h of heat stress, with the highest expression level at 48 h. The expression of *PHO2* expression in roots and shoots was always downregulated, with the highest decrease at 12 h and 24 hour in roots and shoots. The *PHT1;6* expression in shoots shows the highest decrease in expression at the 12 h time point (Supplementary Figure S1M). In the case of other shoot-expressed Pi-related genes, the expression remains almost at the same level. The only exception is present at the 48 h time point for *NLA, SPX-MFS*, and *PHR1* (also 12 h) genes, which are downregulated. *IPS1* expression is almost at the same level during the first eight hours but later decreased. Transcriptomic data revealed that in shoots there were no *PHT1;1, PHT1;4* genes expression; *PHO1* expression was also very low compared to the root expression. That is why even subtle changes in *PHO2, PHT1;6* genes expression could influence Pi concentration. At 1 and 12 h time-points we observed that both these genes had been affected by heat stress. At 1 h time-point transcriptomic data showed significant decreased expression of *PHO2*. RT-qPCR confirmed the transcriptomic data but probably the fold change was too low to be significant in RT-qPCR analysis. Interestingly RT-qPCR showed significant increased *PHT1;6* gene expression and it could be coupled with an increased Pi level (**Figure 5B**). At 12 h time-point we observed significant decreased expression of *PHO2* and *PHT1;6*. Previously we noted that decreased level of *PHO2* gene in barley led to an increase of Pi concentration (Pacak et al., 2010). Further studies on protein level as well as identification of the PHO2 “partner proteins” in shoots are necessary to reveal the role of PHT1;6 in Pi concentration homeostasis in shoots. We were not able to detect mature miR399 in barley shoots grown in heat stress and control conditions (**Figure 6A**). The expression of the miR827 fluctuates in control conditions, and these fluctuations are also observed in heat-treated plants (**Figure 6B**). This observation allowed us to draw the conclusion that the changes in *PHO2* and *SPX-MFS* gene expression in barley shoots (their mRNAs are targeted by miR399 and miR827, respectively) result from transcriptional rather than post-transcriptional regulation. Surprisingly, the observed gene-expression changes in roots do not affect the final root Pi concentration. We noted a significant Pi concentration increase at the 1- and 12-h time-points of heat stress in barley shoots, but not at other time-points (**Figures 5A,B**). This data shows that barley plants exposed to heat stress modulate the expression level of Pi-related genes to stabilize Pi concentration. Although the Pi concentration at the end of 48 hours of heat stress was similar to that of the control plants, heat stress inhibited barley growth and leaves no 4 development (**Figures 7A** and **8**). The leaves of barley plants are shorter one week after the end of the heat stress period (leaf number four), and we observed faster leaf senescence (leaf number one, **Figure 8**). Growth impairment and premature senescence may result from the macronutrient homeostasis disturbances resulting from heat-stress-affected gene-expression regulation.

### Dynamic Changes in Expression Pattern of Barley Pi-Related Genes upon Phosphate Starvation and Pi Re-supply

Analysis of Pi-related barley gene expression under heat stress revealed fast and dramatic changes at the transcriptional level, which finally led to the stabilization of Pi concentration in the plant tissues. To learn more about the regulation and pattern of expression of Pi-related genes in barley, we studied the same set of genes for their expression pattern in roots under control conditions, Pi starvation, and Pi re-supply. Plants were grown at P-limiting conditions and then exposed to P sufficiency. Time-course changes in gene expression were compared to the changes in parallel sets of plants maintained at P-limiting or P-sufficient conditions (control conditions).

**FIGURE 9:**
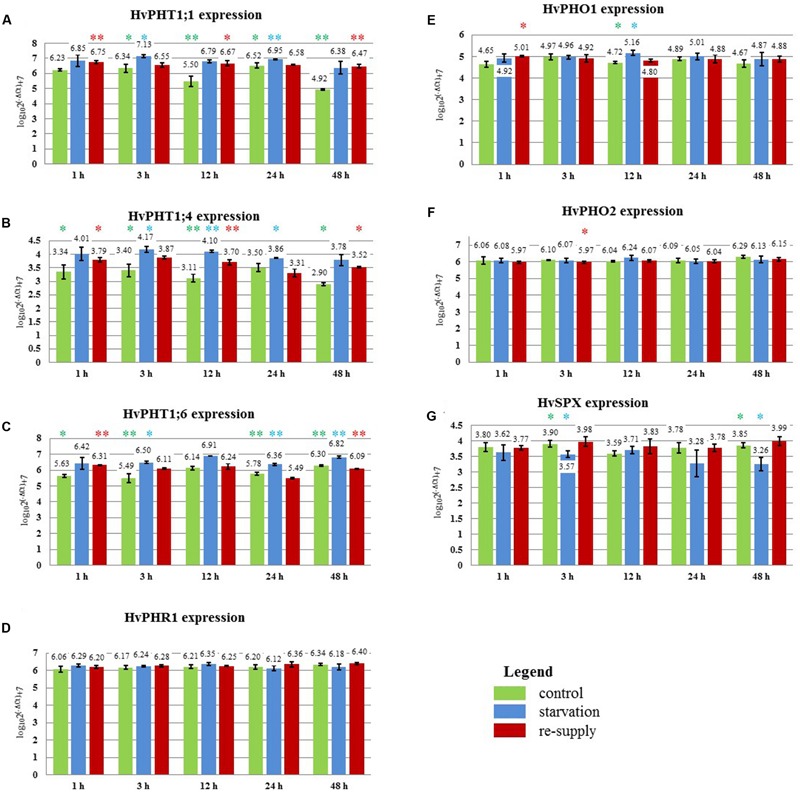
**Barley cv. Rolap Pi-related gene expression under different Pi regime: control, Pi-starvation and Pi re-supply conditions.** RT-qPCR analyses was performed for the expression pattern of the following genes: **(A)** *PHT1;1*, **(B)** *PHT1;4*, **(C)** *PHT1;6*, **(D)** *PHR1*, **(E)** *PHO1*, **(F)** *PHO2*, **(G)** *SPX-MFS*. The expression levels were analyzed + 1, + 3, + 12, + 24, + 48 h after Pi re-supply. Two, 2-week old barley plants per pot represented one sample; three samples were analyzed at each time point and treatment. Colored stars represents statistical significance of the gene expression differences: green ^
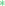
^ – differences between control and low Pi plants; blue ^
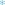
^ – differences between low Pi and Pi re-supplied plants; red ^
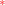
^ – differences between control and Pi re-supplied plants. ^∗^*p* < 0.05, ^∗∗^*p* < 0.005.

In 2-week old barley cv. Rolap *PHT1;1, PHT1;4*, and *PHT1;6* showed increased expression under low Pi conditions compared to the control conditions. Pi-resupply down-regulated Pi transporters expression what was observed after 3 h of the Pi re-supply (**Figure 9**). Especially *PHT1;6* gene was sensitive to the Pi re-supply. Its expression after 48 h was even lower in Pi re-supplied plants compared to the control ones (**Figure 9C**). After 48 h of the Pi-resupply *PHT1;1* gene expression was not distinguishable from plants grown under low Pi. During heat stress we observed that *PHT1;1* gene was the most affected *PHT1* transporter gene by heat stress. It showed plant flexibility in gene expression regulation upon different stresses in order to control Pi homeostasis. Among two genes which mRNA levels are controlled by microRNA, *SPX-MFS* showed higher sensitivity to two abiotic stresses applied. It was constantly downregulated during heat stress and up-regulated after Pi-resupply at 3 h and 48 h time-points (**Figure 9G**). *PHO2* expression was even lower in Pi re-supplied plants compared to the control ones (**Figure 9F**). It showed that in the experimental conditions Pi did not influence *PHO2* expression. There was no effect of Pi concentration on *PHR1*. Compared to heat stress we observed different *PHO1* expression change (**Figure 9E**). During heat stress at 1 h time point *PHO1* expression was down-regulated but after Pi re-supply its expression was even increased compared to the control plants. It could be explained by the fact that plants produced more PHO1 protein to transport available Pi from roots to shoots. After 12 h *PHO1* expression in Pi re-supplied plants reached the control level. The weight of barley plants from low Pi conditions was 62% of the control plants weight. The phenotypes of the Pi control, Pi limitation, Pi re-supplied plants are presented in **Figure 7B**.

**FIGURE 10:**
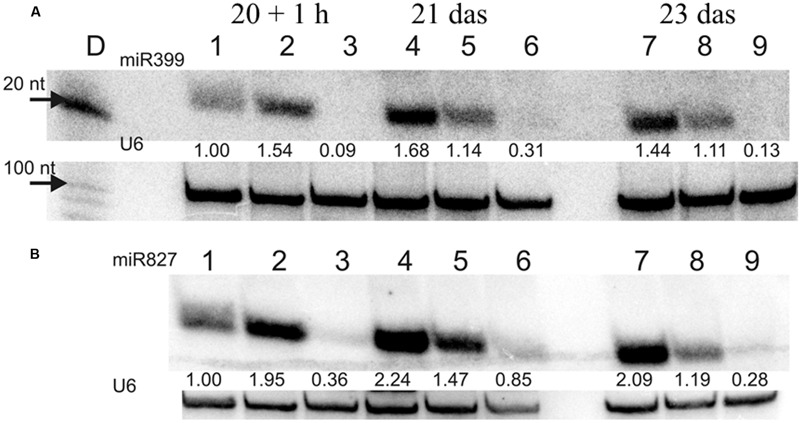
**(A)** miR399 and **(B)** miR827 expression levels in barley roots. Northern blot was performed with probes against either miR399c or miR827. 1, 4, 7 – plant root material from low Pi concentration culture; 2, 5, 8 – plant root material from Pi re-supply conditions; 3, 6, 9 – plant root material from control conditions. U6 was used as RNA loading control, values below the miR bands represent calculated miR expression level normalized to U6 expression.

**FIGURE 11:**
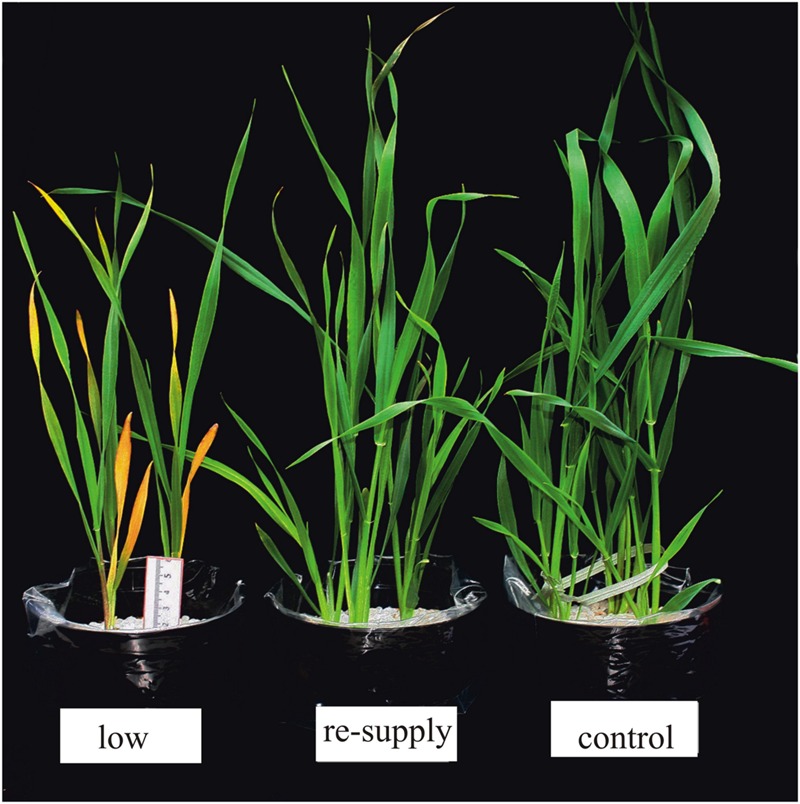
**Twenty-three-day-old barley *cv*. Black Hulless plants grown under different Pi regime in soil with low Pi concentration, after Pi re-supply and under control conditions**.

In our previous work we silenced *PHO2* gene using VIGS approach and observed Pi concentration increase in barley shoots and the *PHO2* downregulation in barley roots (Pacak et al., 2010). For this purpose we used Black Hulless barley cultivar. We used the same cultivar to analyze Pi-related gene expression under different Pi regime. Moreover we kept plants longer (23 days) in low Pi condition to observe more remarkable differences in Pi-related genes expression. Root material was collected at 10, 15, 20 das +1 h after Pi re-supply, 21, and 23 das. RNA was isolated from root tissue, and expression was analyzed for a set of Pi-related genes. The greatest differences in gene expression was observed between plants grown under P-sufficient and P-limiting conditions for *IPS1, PHT1;1*, and pri-miR399c. At control conditions, the highest decrease ranged from 1349-, 447-, and 347-fold reduction, respectively, as compared to Pi-starvation condition (Supplementary Figures S2A,K,L). After Pi re-supply, we observed a weaker reduction of *IPS1, PHT1;1*, and pri-miR399c (which was 26-, 35-, and 44-fold, respectively). These reduction levels were similar to those observed for *PHO1* (33-fold, 24 h) and *PHT1;1* (20-fold, 12 h) during the heat stress (Supplementary Figure S1B,K). Interestingly, *IPS1* expression was downregulated after Pi re-supply but upregulated 11-fold (48 h) after heat stress (Supplementary Figures S1G and S2L). Expression of the *PHO2* and *SPX-MFS* decreased in Pi-limited plants at the early steps of development; but later, their expression reached the same levels as in the P-fed controls (Supplementary Figures S2G,I). The expression of *PHO1* is not changed significantly in different Pi-regime conditions in barley roots. During heat stress both *PHT1;1* and *PHO1* genes expression is downregulated, but Pi concentration affects only the *PHT1;1* expression. No reduction of *PHO1* expression may prevent Pi-over accumulation after Pi re-supply treatment. The higher expression of pri-miR399c at the early stages of development correlated with the lower expression of the *PHO2* in Pi limited (compared to control plants). At later growth stages, the expression of *PHO2* approached the level in the P-sufficient controls plants, although the abundance of pri-miR399c and mature miR399 was still on a high level in Pi starvation condition (Supplementary Figure S2K and **Figure 10A**). This suggests that the role of miR399 in *PHO2* gene-expression regulation is not crucial at the latter developmental stages. Accordingly, Pi re-supply did not change the level of *PHO2* expression significantly, although the expression of pri-miR399 and, consequently, mature miR399 was strongly decreased (Supplementary Figure S2K and **Figure 10A**). Like the mature miR399, miR827 also showed a reduced level after Pi re-supply, corresponding to a reduction to 57% at 3 days after Pi re-supply compared to the P-limiting plants (**Figure 10B**). Pi re-supply resulted in strongly increased Pi concentrations in barley roots (Supplementary Figure S2M), but the reached level was similar to the Pi concentration level in the control plants. Harvested shoot weights were suppressed by Pi limitation already by 15 das, representing only 40% of the weight of the P-sufficient control plants at 23 das (**Table 3**). The re-supply of P-to-P limited plants at 20 das produced a marked growth response over the subsequent three days. Symptoms of early senescence were observed for the older leaves of P-limited plants (**Figure 11**).

**Table 3:** The weight of control, low Pi, and Pi-supply conditions growing plants.

Harvest time	Shoot fresh weight (g/pot)		
	Control plants	low Pi	Pi re-supply
10 das	1.33 ± 0.06	1.17g ± 0.15	–
15 das	3.67 ± 0.45	2.53 ± 0.38	–
20 das+1 h	8.73 ± 1.24	4.57 ± 0.32	5.67 ± 0.67
21 das	11.10 ± 0.46	4.43 ± 0.29	7.60 ± 0.53
23 das	14.77 ± 1.55	5.80 ± 4.16	11.43 ± 1.69

*Values are means ± SD; *n* = 3.*

## Discussion

Studies of the expression of phosphate-related genes in crops are crucial to understand the complexity of adaptive reactions in plants grown under phosphate depletion (Baker et al., 2015). This is especially important, due to the fact that phosphate rock for fertilizer production is a limited resource that requires careful management, since it is estimated that all cheap forms of phosphorus will soon be exhausted (Cordell and White, 2014). It is known that heat stress has a big impact on plant growth. The warming climate is one of the biggest challenges for agriculture, as one degree increase in temperature may decrease crop productivity by as much as 10% (Zinn et al., 2010). Heat stress inhibits plant functions in many ways. In barley, it reduces tillering and decreases plant height and spike length (Abou-Elwafa and Amein, 2016). This work shows that a heat-stress period influenced barley morphology such that, after one week, specific leaves were shorter than the corresponding leaves of control plants, and the oldest leaf showed more severe senescence (**Figure 8**). Heat stress affects the expression of many genes, including those involved in calcium and sugar signaling in wheat, TFs (Hsf, NAC, AP2/ERF, WRKY, MYB, and C_2_H_2_) in rice panicles, or six different peroxidases in switchgrass (Qin et al., 2008; Zhang et al., 2012; Li et al., 2013; Banerjee and Roychoudhury, 2015). The predicted increasing exposure of crop plants to heat stress requires an understanding of how they regulate macronutrient homeostasis under such circumstances. We have shown that heat stress affects the expression of Pi-related genes. This is especially visible in root tissues. Already after one hour of heat stress, we observed decreased levels of *PHT1;1, PHT1;4*, and *HvPHT1;6*, which should result in a decreased level of Pi concentration in roots. However, Pi concentration did not change. This could be explained by the reduced expression levels of *PHO1, PHO2*, and *SPX-MSF* genes. The *pho1* mutant in *Arabidopsis* is defective in Pi transport from root to shoot, resulting in a strong Pi deficiency in shoot tissue (Stefanovic et al., 2011). On the other side, *PHO1*-gene over-expression in *Arabidopsis* leaves led to the increased export of Pi into the xylem vessels (Stefanovic et al., 2011). Barley *PHO1* gene expression is weakly responsive to Pi starvation (similar to *Arabidopsis*). The heat-stress impact on *PHO1* gene expression shown here reveals that this gene represents an important Pi homeostasis regulatory element (Hamburger et al., 2002). This data may explain why Pi concentration in barley roots is non-sensitive to heat stress. We assume that Pi transport to the shoots is inhibited during heat stress. The observed decrease of the *PHO2* transcript level in roots may be responsible for the lower degradation of Pi transporters. Interestingly, we observed that *PHT1;4* gene expression increased during heat stress, which could compensate for the decrease of *PHT1;1* expression.

After one hour of heat stress, all analyzed Pi-related genes in the shoots have stable expression compared to the roots, with only one exception: *PHO2* expression was downregulated significantly after one hour of heat treatment. Previously, we observed that the silencing of the *PHO2* gene resulted in an increased Pi concentration (Pacak et al., 2010). Interestingly, *PHR1* and *PHR2* TFs expression was not changed in either barley roots or shoots. Since *PHR1* expression is stable, other TFs can be responsible for either the downregulation or upregulation of Pi-related genes during heat stress. In roots *PHR2* expression is downregulated in the further time-points.

In further experiments, we analyzed plants grown in soil with a different Pi supply. *HvIPS1, HvPHT1;1, HvPHT1;4, HvPHT1;6*, and *MIR399c* showed a clear response to Pi starvation. After Pi replenishment, we observed a reduction of *HvPHT1;1, HvPHT1;4, HvPHT1;6, HvIPS1*, and pri-miR399c expression levels. In contrast to the heat stress Pi re-supply treatment did not downregulate *PHO1* gene expression (with one exception 12 h time-point, barley cv. Rolap). We conclude that barley plants utilized two strategies to regulate Pi concentration. Upon heat stress both *PHT1;1* and P*HO1* genes are downregulated and Pi concentration is stable. Pi re-supply treatment downregulated *HvPHT;1* expression but not *PHO1*. Its expression is even higher at + 1 h time-point (**Figure 9E**). This allows for Pi concentration increments but prevent Pi over-accumulation. In Black Hulless 10- and 15-day-old plants, we observed higher *HvPHO2* expression in plants grown in a high Pi supply (compared to low Pi conditions). The data showed that *HvPHO2* expression was influenced by the Pi content in soil and correlated with an increase in pri-microRNA399c expression. *PHO2* expression was especially affected by the concentration of Pi in the first two weeks of plant growth. MiR399 and miR827 are the only microRNAs that bind to the 5′ UTR of mRNAs in *Arabidopsis* (Ding et al., 2012). Their levels increased highly during Pi starvation and also decreased after Pi supply. The early senescence of leaf no. 1 of barley plants grown in low Pi suggests the remobilization of Pi from this leaf to the other plant parts.

It was previously shown that, at an ambient temperature (23°C), miR399a, b, and d are more abundant by 1.30–1.31-fold than at 16°C (Lee et al., 2010). During heat stress at a higher temperature (35.5°C), we observed the expression of *PHO2* to be downregulated in both roots and shoots. Our Northern analysis of the shoot samples did not show any miR399 signals. The low abundance of miR399 and miR827 in Pi-sufficient conditions and after heat treatment was not surprising. The abundance of these microRNAs is elevated during Pi starvation, and other stresses (like drought) in *Brachypodium distachyon* are not sufficient to induce their expression (Bertolini et al., 2013). We concluded that, apart from the post-transcriptional action of miR399 on *PHO2* expression, downregulation may be caused on the transcriptional level. We performed *PHO2*-promoter analysis and found binding sites for several TFs. Some of them are presented in **Table 4**.

**Table 4:** Motifs present in the barley *PHO2* promoter.

motif	recognized by	position within promoter
CArG	MADS-box TF	-1299, -1290
CGT[G/A]	NAC TF core binding signals	five motifs within 1422 nt promoter sequence
RYCGAC	CBF (C-repeat (CRT) binding factors)	-913; -908
GCCGAC, DRE/CRT core, dehydration-responsive element/C-repeat	DREB1A	-913; -908

**FIGURE 12:**
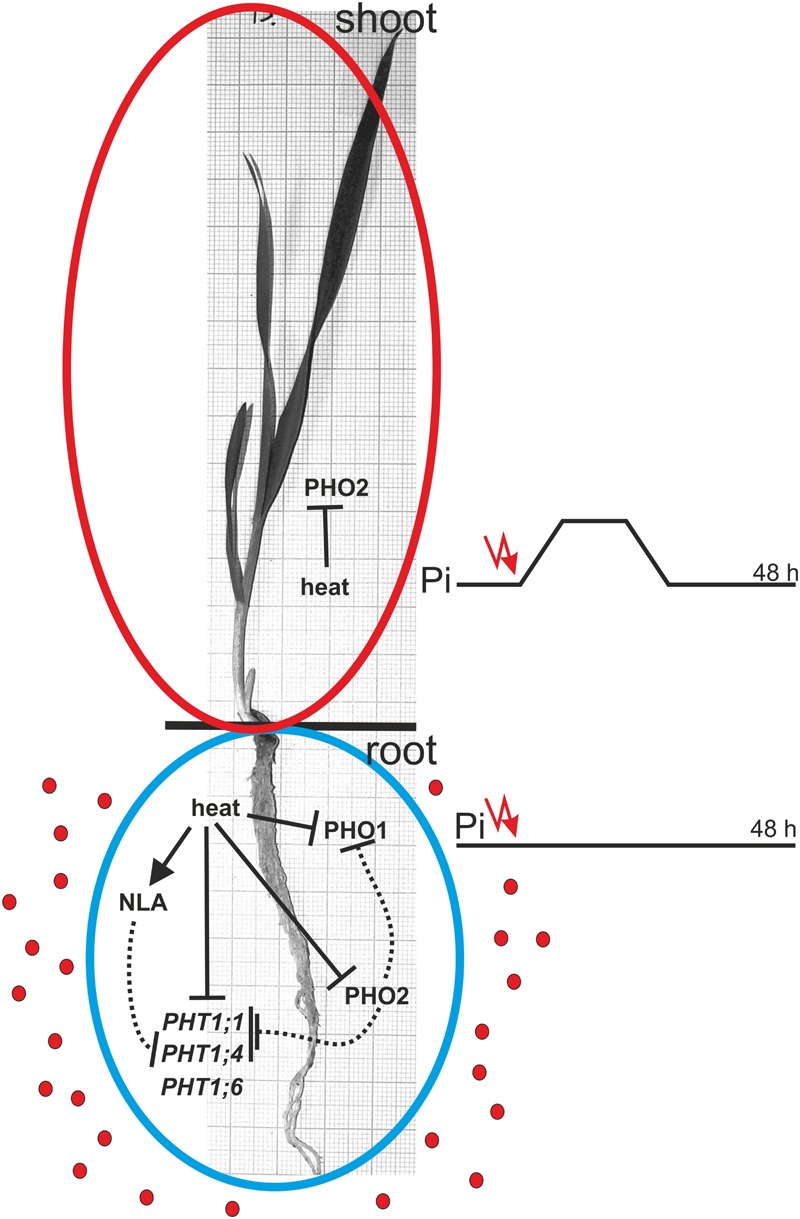
**Model of heat stress impact on Pi-related genes expression and their Pi-homeostasis-related activity.** The solid and dotted lines represent regulation on the transcriptional and protein levels, respectively. Red dots represent Pi ions.

Our data suggests that heat stress affects the expression of barley Pi-related genes with an impact on plant Pi concentration during the first period of stress. Surprisingly, remarkable changes in the gene expression after 24 and 48 h of heat stress did not result in changed Pi concentrations in the barley plants. The model of gene action during heat stress is presented in **Figure 12**. Generally, we observed two strategies applied by barley plants to cope with heat stress and Pi-supply treatment. During heat stress, the expression of *PHT1* transporters (especially *PHT1;1*) significantly decreased, with a simultaneous reduction of *PHO1* expression, thus blocking Pi eﬄux from roots to shoots. This stabilizes the Pi concentration in barley roots. During the Pi re-supply treatment, *PHT1;1* expression is also reduced (but not *PHO1*). This reduction is in response to Pi accessibility, but it did not hamper the increase of Pi concentration in the root. One could expect that the increased Pi concentration in roots should be accompanied by the incremental expression of *PHO1*. Later, after three days of Pi re-supplementation, the expression of the *PHO1* gene remains stable.

Further studies on barley Pi-homeostasis maintenance during heat stress are necessary, mainly at the protein and RNA levels. For example, it was recently shown that *cis* -NAT_PHO1;2_ long non-coding RNA promotes PHO1;2 translation (Secco et al., 2010; Jabnoune et al., 2013). Previously it was reported that *NLA* gene involved in adaptive responses to low nitrogen conditions in *Arabidopsis* is regulated by Pi-starvation induced miR827 (Kant et al., 2011). There are more connections between nitrogen and phosphate metabolism pathways. *Arabidopsis* mutants *phf1* and *pht1;1* were identified as suppressors of the *nla* mutant. These suppressors restore the *nla* mutant phenotype to WT (Kant et al., 2011). Thus the observed decreased level of the *PHT1;1* expression during heat stress could be connected to the *NLA* gene expression up-regulation.

Studies presented in this work shed a new light on the mechanisms of Pi-homeostasis regulation and can help to improve crop plants in their response to unfavorable changes in the environment.

## Author Contributions

AP designed, performed, analyzed all experiments, prepared manuscript, tables, and figures, wrote manuscript, MB-P performed Pi concentration analysis and analyzed experiments, AS-B designed, performed heat stress experiment, KK designed, performed heat stress experiment, PS performed Pi concentration analysis, KM analyzed transcriptome data, IJ designed Pi re-supply experiment and assisted in manuscript preparation, AJ assisted in manuscript writing, editing, ZS-K designed heat experiment, wrote manuscript.

## Conflict of Interest Statement

The authors declare that the research was conducted in the absence of any commercial or financial relationships that could be construed as a potential conflict of interest.

## Footnotes

1 http://plants.ensembl.org/Hordeum_vulgare/Info/Index

2 http://www.ncrnalab.dk/#rnafolder/rnafolder.php

3 http://webblast.ipk-gatersleben.de/barley/
