# Supplementary material for: Heat Stress Affects Pi-related Genes Expression and Inorganic Phosphate Deposition/Accumulation in Barley
Source: Front Plant Sci. 2016 Jun 24;7:926. doi: 10.3389/fpls.2016.00926 (PMC4919326; doi:10.3389/fpls.2016.00926)
Supplement: Supplementary file 1 [file Image_1.PDF]

frontiers

Supplementary Material 1

Heat stress affects Pi-related genes expression and inorganic phosphate deposition/accumulation in barley

Andrzej Pacak*, Maria Barciszewska-Pacak, Aleksandra Swida-Barteczka, Katarzyna Kruszka, Pawel Sega, Kaja Milanowska, Iver Jakobsen, Artur Jarmolowski, Zofia Szweykowska-Kulinska

*** Correspondence**: apacak@amu.edu.pl

1 Supplementary Data

Supplementary Material should be uploaded separately on submission. Please include any supplementary data, figures and/or tables.

Supplementary material is not typeset so please ensure that all information is clearly presented, the appropriate caption is included in the file and not in the manuscript, and that the style conforms to the rest of the article.

2 Supplementary Figures and Tables

For more information on Supplementary Material and for details on the different file types accepted, please see here.

2.1 Supplementary Figures

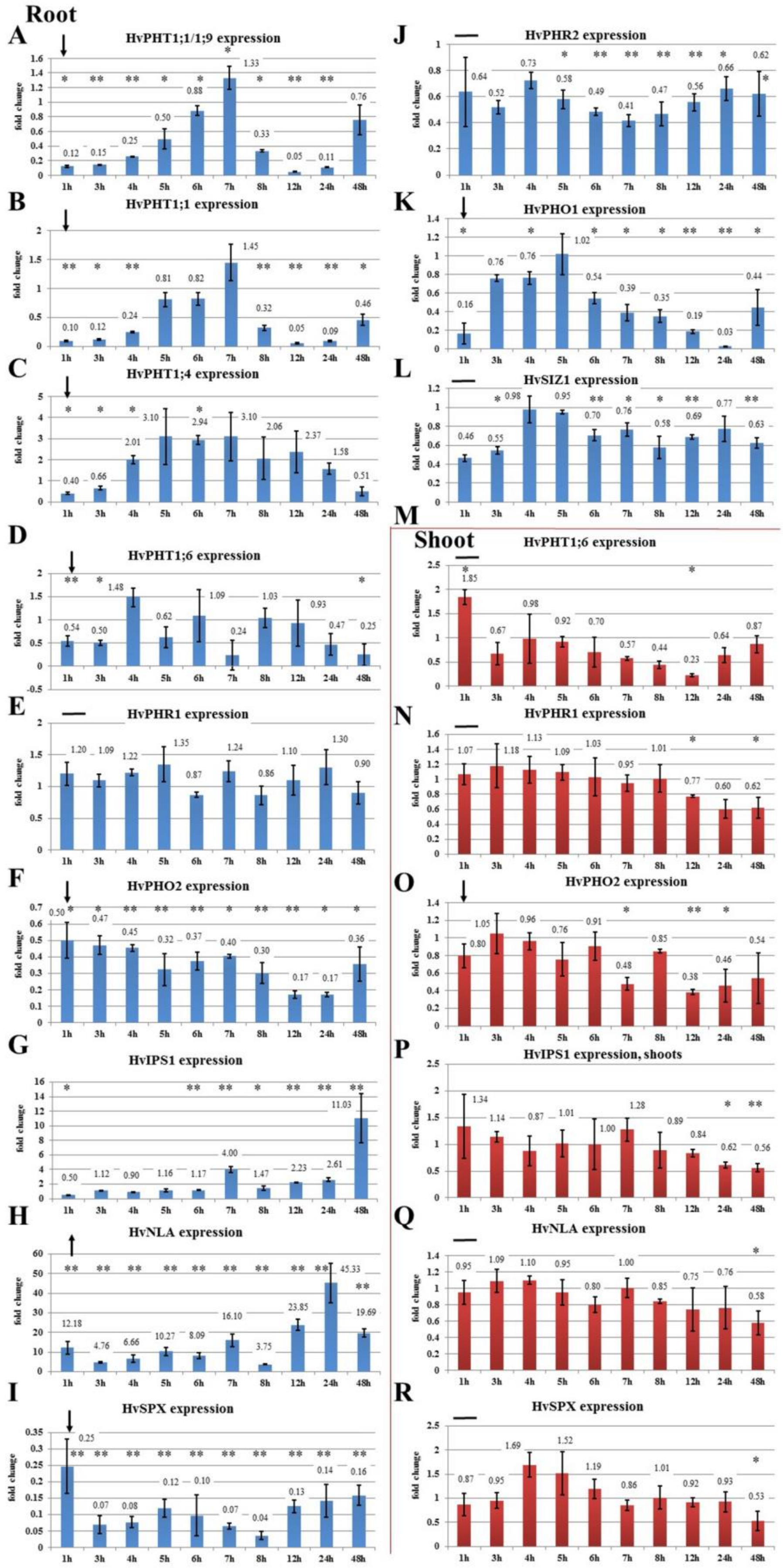
Root
A HvPHT1;1/1;9 expression
B HvPHT1;1 expression
C HvPHT1;4 expression
D HvPHT1;6 expression
E HvPHR1 expression
F HvPHO2 expression
G HvIPS1 expression
H HvNLA expression
I HvSPX expression
J HvPHR2 expression
K HvPHO1 expression
L HvSIZ1 expression
M
Shoot
HvPHT1;6 expression
N HvPHR1 expression
O HvPHO2 expression
P HvIPS1 expression, shoots
Q HvNLA expression
R HvSPX expression
fold change
1h 3h 4h 5h 6h 7h 8h 12h 24h 48h

Supplementary Figure 1. Barley (cv. Rolap) Pi-related gene expression under heat stress. RT-qPCR analyses was performed for the expression pattern of the following genes: root **(A)** *PHT1;1/1;9*, **(B)** *PHT1;1*, **(C)** *PHT1;4*, **(D)** *PHT1;6*, **(E)** *PHR1*, **(F)** *PHO2*, **(G)** *IPS1*, **(H)** *NLA*, **(I)** *SPX-MFS*, **(J)** *PHR2*, **(K)** *PHO1*, **(L)** *SIZ1*; shoot **(M)** *PHT1;6*, **(N)** *PHR1*, **(O)** *PHO2*, **(P)** *IPS1*, **(Q)** *NLA*, **(R)** *SPX-MFS*. The expression levels were analysed at + 1, + 3, + 4, + 5, + 6, + 7, + 8, + 12, + 24 and + 48 hours after heat application. Two, 2-week old barley plants per pot represented one sample; three samples were analysed at each time point and treatment. * - p-value < 0.05, ** - p-value < 0.005. Up- and down-directed arrowheads represent up- or down-regulated genes expression, respectively; horizontal line represents no significant difference in gene expression shown by transcriptomic analysis at 1 h time-point. Blue and red bars represent fold change in root and shoot tissue, respectively.
