# Supplementary material for: Heat Stress Affects Pi-related Genes Expression and Inorganic Phosphate Deposition/Accumulation in Barley
Source: Front Plant Sci. 2016 Jun 24;7:926. doi: 10.3389/fpls.2016.00926 (PMC4919326; doi:10.3389/fpls.2016.00926)
Supplement: Supplementary file 2 [file Image_2.PDF]

Supplementary Material Figure 2

Heat stress affects Pi-related genes expression and inorganic phosphate deposition/accumulation in barley

Andrzej Pacak*, Maria Barciszewska-Pacak, Aleksandra Swida-Barteczka, Katarzyna Kruszka, Pawel Sega, Kaja Milanowska, Iver Jakobsen, Artur Jarmolowski, Zofia Szweykowska-Kulinska

*** Correspondence:** apacak@amu.edu.pl

1 Supplementary Data

Supplementary Material should be uploaded separately on submission. Please include any supplementary data, figures and/or tables.

Supplementary material is not typeset so please ensure that all information is clearly presented, the appropriate caption is included in the file and not in the manuscript, and that the style conforms to the rest of the article.

2 Supplementary Figures and Tables

For more information on Supplementary Material and for details on the different file types accepted, please see here.

2.1 Supplementary Figures

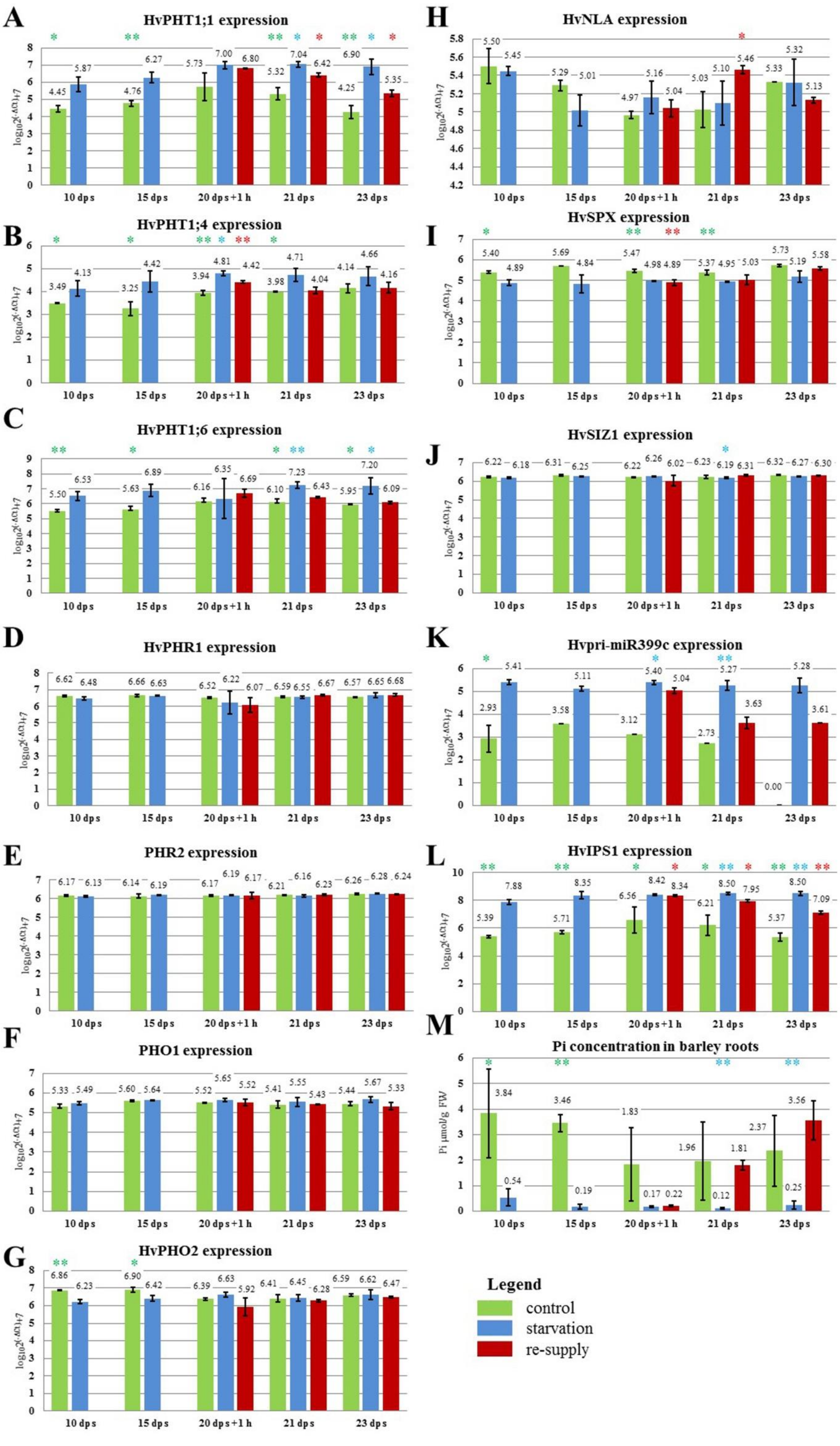
A
HvPHT1;1 expression
B
HvPHT1;4 expression
C
HvPHT1;6 expression
D
HvPHR1 expression
E
PHR2 expression
F
PHO1 expression
G
HvPHO2 expression
H
HvNLA expression
I
HvSPX expression
J
HvSIZ1 expression
K
Hvpri-miR399c expression
L
HvIPS1 expression
M
Pi concentration in barley roots
Pi μmol/g FW
10 dps
15 dps
20 dps+1 h
21 dps
23 dps
Legend
control
starvation
re-supply

Supplementary Figure 2. Barley (cv. Black Hulless) Pi-related gene expression under different Pi regime: control, Pi-starvation and Pi re-supply conditions. RT-qPCR analyses was performed for the expression pattern of the following genes: **(A)** *PHT1;1*, **(B)** *PHT1;4*, **(C)** *PHT1;6*, **(D)** *PHR1*, **(E)** *PHR2*, **(F)** *PHO1*, **(G)** *PHO2*, **(H)** *NLA*, **(I)** *SPX-MFS*, **(J)** *SIZ1*, **(K)** pri-miR399c, and **(L)** *IPS1*. **(M)** Pi concentration in roots of plants grown in soil with control Pi concentration (green bars), low Pi (blue bars), with Pi re-supply (red bars). The expression levels were analysed 10, 15, 20 + 1 hour, 21, and 23 days after sowing in root tissue. Three plants per pot represented one sample; three samples were analysed at each time point and treatment. Colored stars represents statistical significance of the gene expression differences: green * - differences between control and low Pi plants; blue * - differences between low Pi and Pi re-supplied plants; red * - differences between control and Pi re-supplied plants. * - p-value < 0.05, ** - p-value < 0.005.
